# Supplementary material for: Ungulate use of locally infectious zones in a re-emerging anthrax risk area
Source: R Soc Open Sci. 2020 Oct 21;7(10):200246. doi: 10.1098/rsos.200246 (PMC7657905; doi:10.1098/rsos.200246)
Supplement: Figure S1 and S2 [file rsos200246supp1.docx]

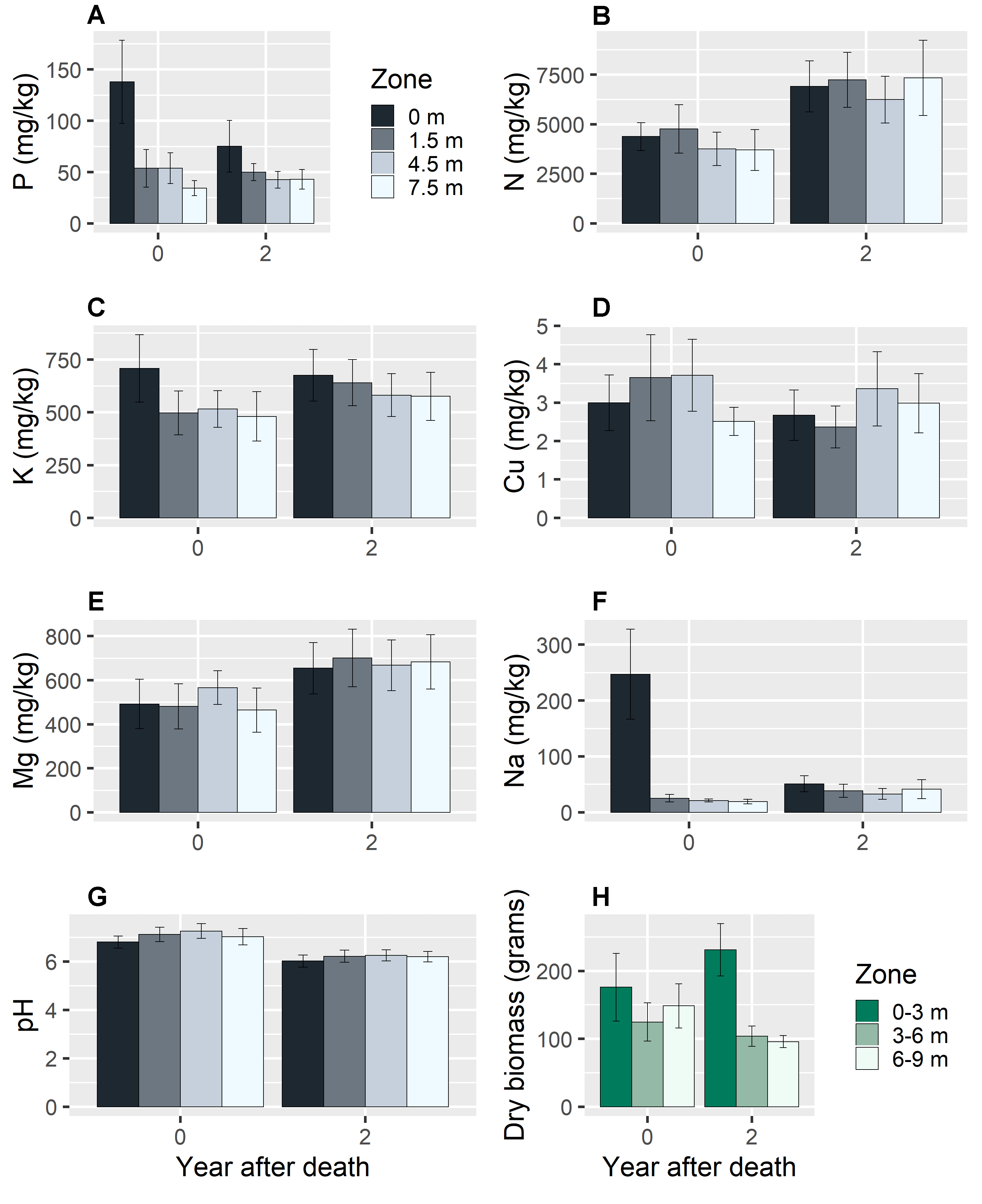
Supplementary Figure 1.  The effects of carcasses on average soil concentrations of P, N, K, Cu, Mg, Na, and pH at four sampling distances (0 m, 1.5 m, 4.5 m, and 7.5 m) and on grass biomass from three sampling zones (0-3 m, 3-6 m, and 6-9 m).


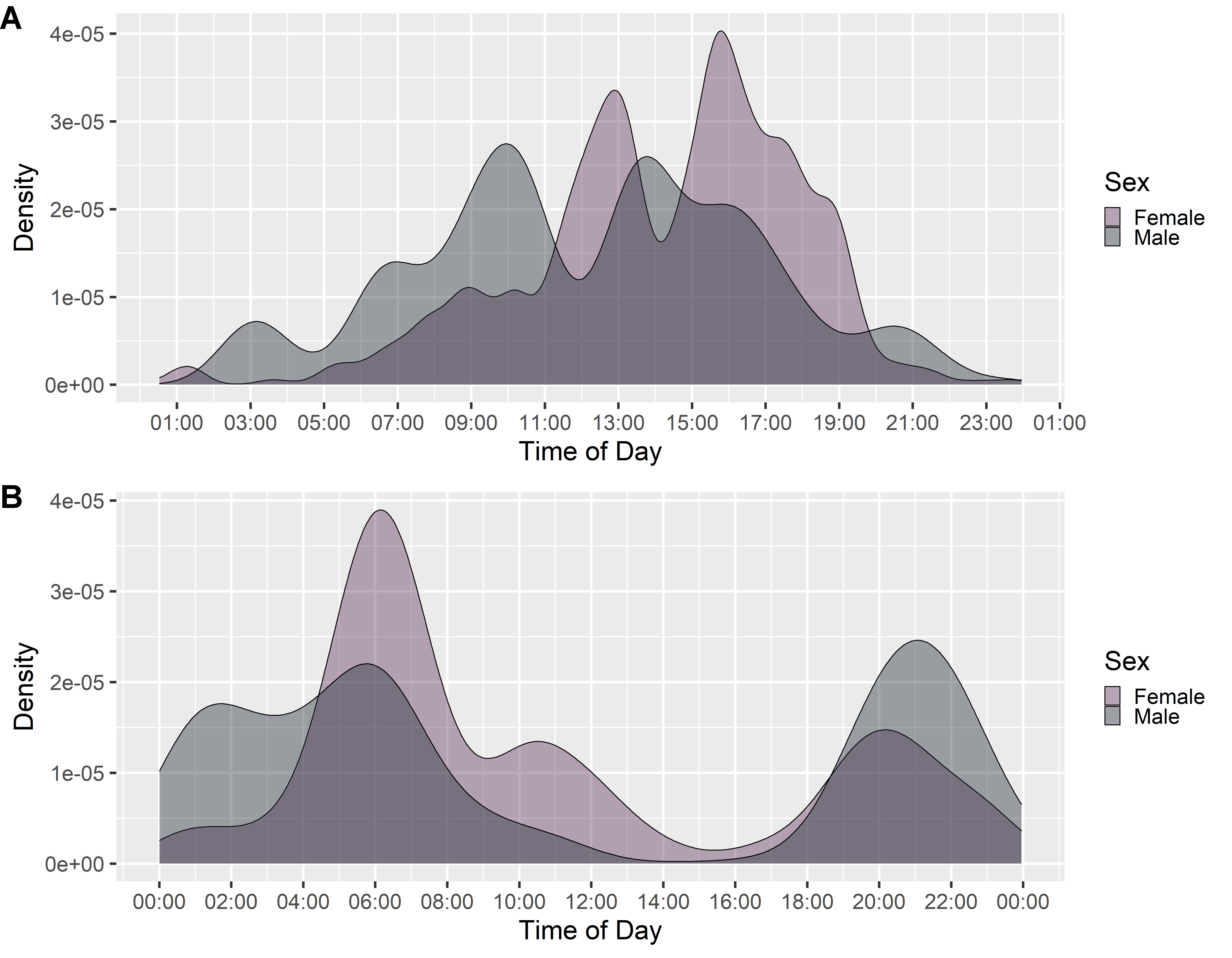


Supplementary Figure 2.  The diurnal activity of bison (A) and elk (B) determined from the time of day when camera traps were triggered by animals.
